# Liquid hyper-absorption as a cause of increased DTPA clearance in the cystic fibrosis airway

**DOI:** 10.1186/2191-219X-3-14

**Published:** 2013-02-27

**Authors:** Timothy E Corcoran, Kristina M Thomas, Stefanie Brown, Michael M Myerburg, Landon W Locke, Joseph M Pilewski

**Affiliations:** 1 Pulmonary, Allergy, and Critical Care Medicine, University of Pittsburgh, UPMC MUH NW628, 3459 Fifth Ave, Pittsburgh, PA 15213, USA; 2 Department of Bioengineering, University of Pittsburgh, Pittsburgh, PA, USA; 3 Department of Chemical Engineering, University of Pittsburgh, Pittsburgh, PA, USA; 4 Department of Cell Biology and Physiology, University of Pittsburgh, Pittsburgh, PA, USA

**Keywords:** Cystic fibrosis, Airway surface liquid, DTPA, Airway permeability

## Abstract

**Background:**

Airway liquid hyper-absorption is a key pathophysiological link between the genetic mutations of cystic fibrosis (CF) and the development of lung disease. Here we consider whether the clearance of radiolabeled diethylene triamine pentaacetic acid (DTPA) might be used to detect changes in airway liquid absorption.

**Methods:**

Tc99m-DTPA was added to the apical (luminal) surface of primary human bronchial epithelial cell cultures from CF and non-CF lungs. Liquid absorption rates were assessed using an optical method and compared to DTPA absorption rates. Measurements of transepithelial electrical resistance (TER) were made to determine the effect of epithelial permeability. DTPA absorption was assessed after stimuli known to influence liquid absorption (volume addition and osmotic gradients) and in cultures containing different proportions of CF and non-CF cells.

**Results:**

DTPA absorption rate was increased in CF cultures matching previous *in vivo* studies in individuals with CF. DTPA and liquid absorption rates were proportional. There was no relationship between TER and DTPA absorption rate when measured in individual cultures. Apical volume addition increased both DTPA and liquid absorption rates. DTPA absorption increased in a dose-dependent manner after basolateral mannitol addition was used to create transepithelial osmotic gradients favoring liquid absorption. Conversely, apical mannitol (a candidate therapy) slowed DTPA absorption in CF cultures.

**Conclusions:**

These results imply that DTPA absorption is directly related to liquid absorption, consistent with increased rates of airway surface liquid absorption in the CF airway, and that modification of liquid absorption from osmotic therapies might be detectable through DTPA absorption measurements *in vivo*.

**Trial registration:**

none

## Background

Cystic fibrosis (CF) is an autosomal recessive disease associated with a single gene - the cystic fibrosis transmembrane conductance regulator (CFTR). Defective function of the epithelial sodium channel (ENaC) also appears to play a key role [1,2]. By controlling Cl^−^ and Na^+^ transport across the apical surface of epithelial cells, CFTR and ENaC establish the osmotic gradients that drive liquid transport across the airway epithelium. Liquid transport must support the hydration of the mucus that lines the airways in order to maintain normal mucociliary and cough clearance in the lungs. The basic defect of CF is associated with a failure to conduct chloride through CFTR. The hyper-absorption of sodium by ENaC has also been described [2]; however, some recent literature disputes this effect [3]. An osmotic gradient that favors the rapid absorption of liquid from the CF airway surface ultimately results in the accumulation of dehydrated mucus in the airways and the gradual failure of mucociliary clearance. CF patients become vulnerable to pulmonary infection which leads to inflammation and, eventually, permanent lung damage, significant losses in pulmonary function, and premature mortality [4-9]. A clinical method for detecting changes in airway liquid absorption could provide a means of screening medications designed to correct the basic defects of CF lung disease. This would include the CFTR correctors and potentiators that have been the focus of recent therapeutic development efforts [10].

Radiolabeled diethylene triamine pentaacetic acid (DTPA) has been used for pulmonary diagnostic imaging for many years. Ventilation scans with aerosolized technetium 99m-labeled DTPA (Tc-DTPA) are commonly performed, and there is extensive literature on the use of DTPA clearance as a measure of lung permeability. Increases in DTPA clearance have been associated with pro-inflammatory stimuli, such as cigarette smoke [11,12], and diseases with inflammatory components, such as asthma [13]. Our previous studies have demonstrated increased rates of DTPA clearance in the lungs of CF patients. Whole-lung clearance rates in these subjects were increased by 27% compared to healthy controls. A multi-probe method (indium 111-DTPA and Tc99m sulfur colloid) was used to estimate the mucociliary and absorptive elements of DTPA clearance in these subjects. The absorptive component of DTPA clearance was increased by more than 30% in the airway-dominated central lung zones in CF [14]. Multiple factors may influence permeability and the clearance of DTPA from the lungs. DTPA is a small charged molecule with no known mechanism for transcellular movement. Tight junction conditions could therefore substantially influence the paracellular absorption of DTPA and its ultimate clearance from the lung. Increases in tight junction diameter by specific cytokine combinations have been demonstrated and may explain increased DTPA absorption in the setting of inflammation [15]. Similarly, the presence of epithelial damage may provide an alternate route for absorption and result in increased DTPA clearance [16]. Mucociliary clearance has been shown to contribute to the clearance of DTPA in the airways [17], and the bronchial circulation rate has also been shown to affect DTPA clearance [18]. Liquid transport through the tight junctions could also influence rates of DTPA absorption. A role for paracellular liquid flux in determining drug absorption rates in the gut has been previously described [19], and osmotic gradients across the epithelium have been shown to substantially influence DTPA absorption rate [20]. Airway liquid absorption can occur through both transcellular and paracellular routes. While the individual contributions of the routes have not been determined, the transcellular path is thought to dominate in CF [21].

Here we have utilized primary human bronchial epithelial (HBE) cells from CF and non-CF lungs to determine whether DTPA absorption might provide a useful gauge of airway liquid absorption. DTPA absorption rates were measured in HBE cultures and compared to liquid absorption rates using an optical method for measuring airway surface liquid (ASL) volume [22]. Mixed-cell cultures including CF and non-CF cells were used to study how the proportional correction of CF pathophysiology affected sodium and chloride currents (*I*_Na+_ and *I*_Cl−_), transepithelial resistance (TER), DTPA absorption, and liquid absorption. Measurements of TER were also performed using a volt-ohm meter to determine the relative effect of tight junction permeability on DTPA absorption from individual cell cultures. We also modulated liquid absorption in the HBE cultures through the use of apical (luminal) volume addition and osmotic gradients and examined the effects of these stimuli on DTPA absorption. Lastly, we assessed the *in vitro* response of DTPA absorption to a well-characterized osmotic therapy using CF HBE cultures.

## Methods

### Human bronchial epithelial cell model

This model provides an *in vitro* representation of the airway epithelium that accurately depicts electrophysiology and CF pathophysiology [21]. Primary HBE cells were isolated from excess airway tissue dissected from lungs removed for transplantation. The protocol for collection was approved by the University of Pittsburgh Institutional Review Board. As previously described [23], the airway sections were digested in a protease solution overnight to detach the epithelial cells from the tissue. The cells were then suspended in epithelial growth media and initially seeded onto sterile tissue culture flasks pre-coated with human placental collagen. After 5 to 6 days, the cells were seeded onto 0.33-cm^2^ collagen-coated transwell filters (0.4-μm pore size, Corning-Costar Transwell Collagen T-cols, Acton, MA, USA) at a density of approximately 2 × 10^6^/cm^2^. When confluent, the cells were maintained at an air-liquid interface, and the basolateral media were changed to differentiation media. All cultures utilized in these experiments were fully differentiated. Each cell line described herein is from a unique donor. Mixed-cell HBE cultures were also prepared including different proportions of CF and non-CF cells. All mixed-cell cultures were prepared from the same CF and non-CF cell lines in the following combinations (CF/non-CF): 100%:0%, 95%:5%, 90%:10%, 75%:25%, 50%:50%, and 0%:100%.

### Ussing chamber measurements

Ussing chamber measurements of epithelial sodium and chloride currents (*I*_Na+_ and *I*_Cl−_, respectively) and TER were performed with each cell line utilized in these experiments, including the mixed-cell cultures. Individual HBE cultures were mounted on Ussing chambers (P2300; Physiological Instruments, San Diego, CA, USA), with custom sliders modified to fit the Transwell inserts, and were continuously short circuited with an automatic voltage clamp (VCC MC8, Physiological Instruments). The bathing Ringer’s solution was composed of 120 mM NaCl, 25 mM NaHCO_3_, 3.3 mM KH_2_PO_4_, 0.8 mM K_2_HPO_4_, 1.2 mM MgCl_2_, 1.2 mM CaCl_2_, and 10 mM glucose. Chambers were constantly gassed with a mixture of 95% O_2_/5% CO_2_ at 37°C, which maintained the pH at 7.4 and established a circulating perfusion bath within the Ussing chamber. Transepithelial resistance was recorded by applying a 10-mV pulse per second via an automated pulse generator. Acquire and Analyze 2.3 (Physiological Instruments) was used to control the voltage clamp and analyze short-circuit current data. Our methods have been previously described in more detail [23]. The amiloride-sensitive *I*_SC_ (*I*_Na+_) was measured by adding 10 μM amiloride to the apical chamber. Following the addition of amiloride, 10 μM forskolin was added, and the Cl^−^ current (*I*_Cl−_) was defined as the decrease in *I*_SC_ following the addition of 100 μM bumetanide to the basolateral surface. Ussing chamber measurements were also performed to determine the effect of 300 mM mannitol on TER. Baseline measurements were made over an approximately 20-min period prior to the addition of 300 mM mannitol to the apical surface, after which time measurements were continued for another 20-min period.

### DTPA absorption measurements

DTPA absorption was measured from 2 to 24 h after the addition of 0.37 MBq/ml Tc-DTPA in Ringer's or phosphate-buffered saline (PBS) solution to the apical (luminal) surface of the HBE cells. Radioactivity was measured in the filter containing the epithelial cells and the ASL by removing it from the media and briefly placing it in a well counter. Results were decay corrected, normalized by starting counts, and fit to exponential curves to generate 24-h percentage clearance rates. We have previously studied the durability of Tc99m binding to DTPA using paper chromatography (Pinestar Technology Red strips, Jamestown, PA, USA) with acetone as a mobile phase to detect free (unbound) ^99m^TcO_4−_. Samples from the mixing vial obtained over 24 h demonstrated <1% free Tc99m. Samples drawn from the apical surface of HBE cells over 24 h after the addition of 10 μL of Tc-DTPA in Ringer’s solution demonstrated 0% to 9% free ^99m^TcO_4−_.

### An optical method to measure ASL volume

**Figure 1:**
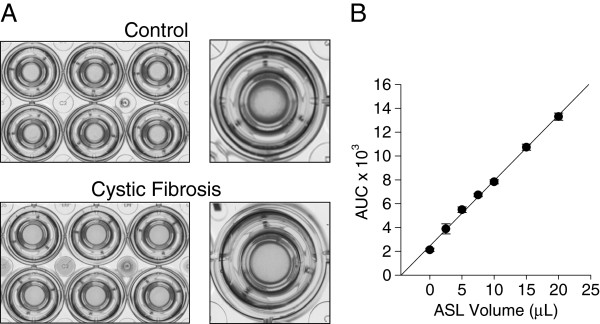
**Optical scanning to measure airway surface liquid volume.** (**A**) Differentiated primary HBE cell cultures were imaged with an optical scanner. As shown in the top panel, non-cystic fibrosis HBE develop a liquid meniscus that surrounds the culture insert. Conversely, this fluid is absent in HBE cultured from cystic fibrosis donors. A magnified view is shown on the right to demonstrate the liquid meniscus that surrounds each individual culture. (**B**) AUC/ASL volume calibration curve. Increasing volumes of Ringer’s solution were applied to the apical surface of differentiated HBE cultures, and the area under the curve was measured as described. Data shown are mean AUC vs. applied ASL volume. *n* = 6.

An optical method was used to measure ASL volume [22]. A meniscus develops at the edge of cultured primary HBE cells and can be visualized by the graded light intensity as shown in Figure 1A. Our method utilizes the refraction pattern induced as transmitted light passes through that meniscus. Plates containing 12 HBE cultures were imaged with an optical scanner (Epson V500, Epson Corporation, Long Beach, CA, USA) and analyzed using an image analysis algorithm in ImageJ (NIH, Bethesda, MD, USA). The light intensity across radial spokes from the center of each filter was measured, and the area under the curve (AUC) was calculated. AUC was integrated into an experimentally determined volume calibration curve as shown in Figure 1B. The relationship between the ASL volume and the AUC is robust and linear across volumes of 0 to 20 μL (*R*^2^ = 0.97).

### Transepithelial electrical resistance measurements

Representative measurements of TER were made in each of the cell lines used in these studies (via Ussing chamber). TER was also measured in a series of individual cell cultures on the day prior to measurements of DTPA absorption, using a volt-ohm meter. Four non-CF lines (30 total cultures) and one CF line (12 cultures) were included. Cultures were submerged in 550 μL of Dulbecco’s modified Eagle’s medium (DMEM), and DMEM was also added to the apical surface (250 μL). After 15 min, three measurements of TER were made for each culture using a Millipore Millicell-ERS Volt-Ohm Meter (Millipore, Billerica, MA, USA). The DMEM was then aspirated, and the cells were returned to the media.

### Effects of apical liquid volume addition on DTPA and liquid absorption

The addition of apical liquid volume increases ENaC current providing a stimulus for liquid absorption [24]. Volumes of 2.5, 5, and 10 μL of Tc-DTPA in Ringer’s solution were added to the apical surface of CF HBE cells (*n* = 6 cultures/case). DTPA absorption and apical liquid volume were then measured over 24 h.

### Effect of transepithelial fluid movement on DTPA absorption

DTPA absorption rates were measured under different osmotic gradients across the epithelium. Tc-DTPA in 10 μL of PBS was used. Two CF and two non-CF cell lines were studied (*n* = 6 cultures per concentration per line). Mannitol (150 and 300 mM) was added to the basolateral media (volume 300 μL). DTPA absorption response to 300 mM apical mannitol was also tested to mimic inhaled therapy in humans [25]. Three CF lines were included (six cultures per case per line).

### Statistical methods

Measurements of *I*_Cl−_, *I*_Na+_, and TER by Ussing chamber and measurements of DTPA absorption rate in CF vs. non-CF cells were compared by unpaired *t* test (averages by line for 9 CF and 11 non-CF cell lines). Comparisons of DTPA absorption after benzamil addition, TER values for individual CF and non-CF cell cultures (made using a volt-ohm meter), DTPA absorption rates with and without apical mannitol, and measurements of TER with Ringer’s vs. Ringer’s + mannitol (by Ussing chamber) were also performed by unpaired *t* test. Linear regression was utilized to determine the relationship between DTPA absorption and liquid absorption (Stata 12, Stata Corp., College Station, TX, USA). For regressions, values for *R*^2^ are presented along with associated *p* values. All values presented in the text are ±standard deviation (SD) unless otherwise indicated. Error bars in figures are ±standard error of the mean (SEM) unless otherwise indicated.

## Results

### DTPA absorption rates in CF and non-CF HBE cell cultures

**Figure 2:**
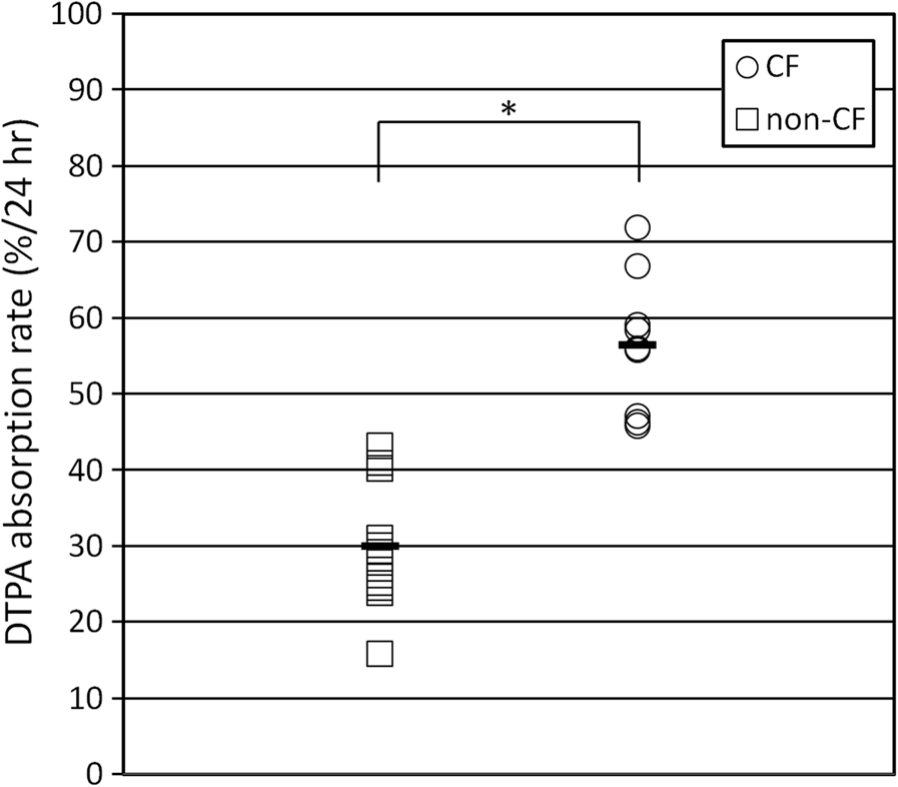
**DTPA absorption rate is increased in epithelial cell cultures from cystic fibrosis lungs.** The mean DTPA absorption rates in well-differentiated cultures from 9 CF and 11 non-CF primary cell lines are shown (six filters averaged for each line). Asterisk, *p* = 0.0013 by *t* test. Measurements were made after the addition of 10 μL of Tc-DTPA in Ringer’s solution.

To determine whether DTPA absorption is different in CF primary airway cells compared to non-CF cells, the retention of DTPA on the apical surface was measured over time. As shown in Figure 2, the mean DTPA absorption rate from the non-CF cell lines was 30.1 ± 8.4%/24 h (11 lines, six cultures per line). The nine CF cell lines absorbed DTPA on average at a significantly higher rate, 56.4 ± 9.1%/24 h (±SD, *p* = 0.0013 by *t* test) (CF cell line genotypes: 4 ΔF508/ΔF508, 2 ΔF508/3659delC, 1 ΔF508/G85E, 1 ΔF508/P.11398S, 1 ΔF508/unknown). Average values of *I*_Cl−_ were 17.5 ± 7.6 μA/cm^2^ for the non-CF lines vs. 0.0 ± 0.5 μA/cm^2^ for the CF lines (*p* < 0.0001); *I*_Na+_, 32.8 ± 19.7 μA/cm^2^ for non-CF vs. 30.4 ± 30.2 μA/cm^2^ for CF (*p* = 0.83); and TER, 350 ± 162 μA/cm^2^ for non-CF vs. 476 ± 405 Ω cm^2^ for CF (*p* = 0.36). Previous studies have demonstrated increased rates of DTPA absorption in the airways of CF subjects [14]. These studies provide *in vitro* corroboration of those results in a setting that excludes infection and the associated inflammation as potential contributory causes.

### Mixed-cell experiments

**Figure 3:**
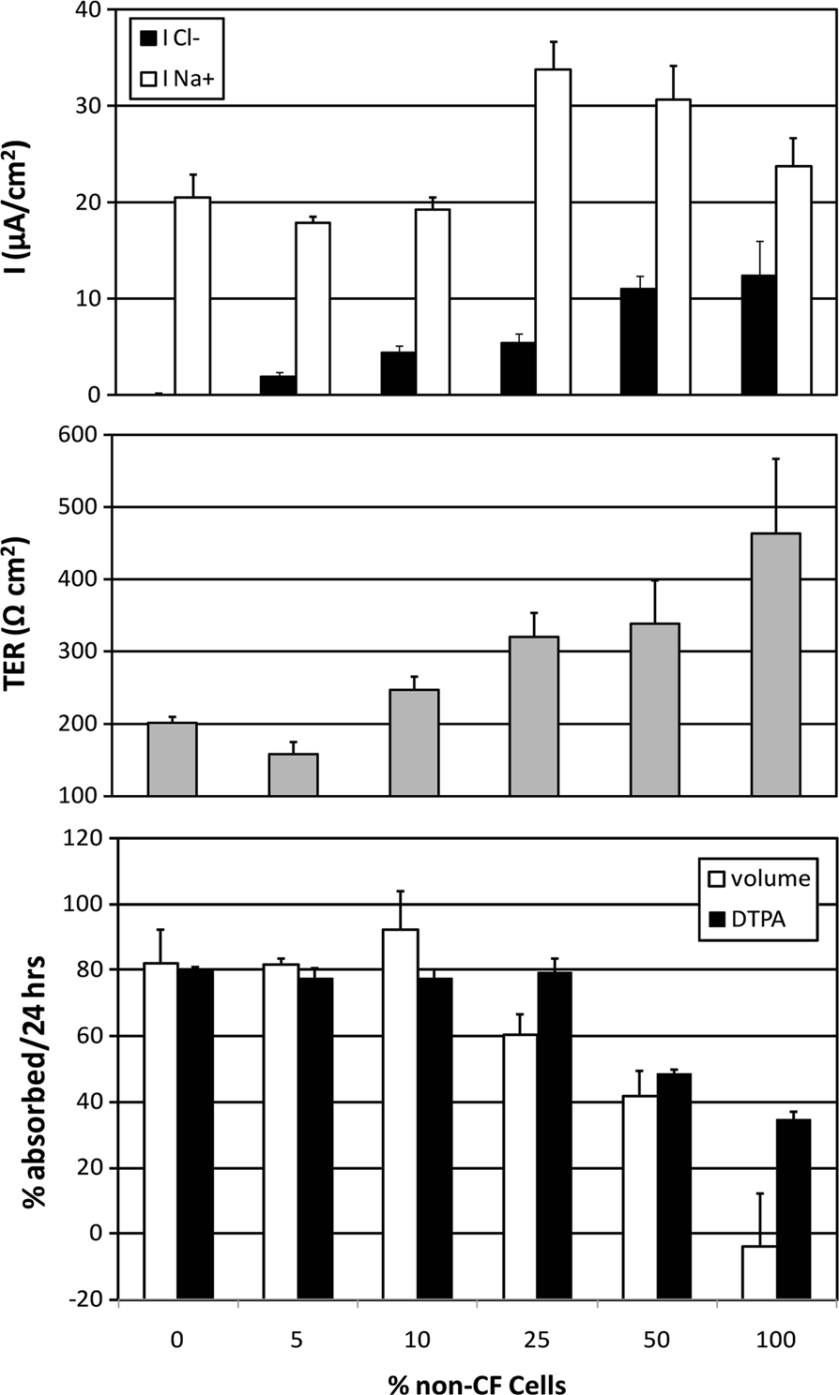
**Mixed-cell experiments and the relationship between *I***_**Cl−**_**, *I***_**Na+**_**, TER, and DTPA and liquid absorption rates.** Mixed-cell HBE cultures were prepared including different proportions of CF and non-CF cells (CF/non-CF): 100%:0%, 95%:5%, 90%:10%, 75%:25%, 50%:50%, and 0%:100%. Data shown are mean *I*_Cl−_, *I*_Na+_, percentage of DTPA absorption at 24 h, and TER values.

The mixing of CF and non-CF cells within individual cultures provides a means of studying how the proportional correction of CF pathophysiology affects epithelial electrophysiology and liquid/DTPA absorption. Figure 3 includes measurements of *I*_Na+_, *I*_Cl−_, TER, and DTPA and liquid absorption rates for a series of mixed-cell cultures. Increases in *I*_Cl−_ and TER are apparent with higher proportions non-CF cells. Both DTPA and liquid absorption decrease with ≥50% non-CF HBEs, following similar patterns. *I*_Na+_ was not correlated with the proportion of CF vs. non-CF cells, DTPA absorption, or ASL absorption. These results suggest that *I*_Cl−_ and TER are the dominant determinants of ASL homeostasis under these experimental conditions. The CF cell line used in this experiment was ΔF508/P.11398S.

### DTPA absorption after benzamil addition

In order to further consider the relationship between *I*_Na+_ and DTPA absorption, we studied DTPA absorption after the addition of 50 mM apical benzamil, a sodium channel blocker vs. a Ringer’s control. DTPA absorption was similar with Ringer’s vs. Ringer’s + benzamil in one CF (ΔF508/3659delC) and one non-CF cell line (*p* = 0.49 and 1.0). In an attempt to further enhance baseline liquid absorption rates, large liquid volumes (~50 μL) were added to the apical surface of another set of cultures on the day prior to measurements of DTPA absorption. DTPA absorption rates with Ringer’s vs. Ringer’s + benzamil in these cells were again similar (two different CF cell lines, both ΔF508/3659delC), *p* = 0.26 and 0.38. Measurements were also made in two non-CF lines. One result approached significance (*p* = 0.06) with decreased DTPA absorption rates associated with benzamil. The other result did not demonstrate a significant difference (*p* = 0.27). This result indicates that changes in *I*_Na+_ do not influence DTPA absorption in this model.

### Comparing DTPA and liquid absorption rates

**Figure 4:**
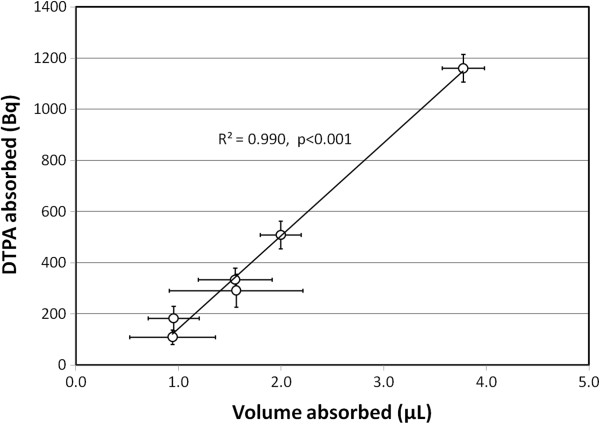
**DTPA absorption is proportional to liquid absorption after volume addition.** The figure compares the radioactive counts of DTPA absorbed to apical liquid volume absorbed at six different time points after the addition of 5 μL of Tc-DTPA in Ringer’s solution (CF HBEs, six cultures included). Liquid volume was measured using a previously described optical technique [22]. Each data point is mean ± SEM. When repeated with different volumes, 2.5 μL: *R*^2^ = 0.96, *p* = 0.003; 10 μL: *R*^2^ = 0.56, *p* = 0.09. When repeated in a different CF cell line, 10 μL: *R*^2^ = 0.90, *p* = 0.004.

Simultaneous measurements of DTPA absorption and ASL volume were performed over a 24-h period after the addition of 5 μL of Tc-DTPA to the apical surface of CF HBEs (ΔF508/3659delC). The volume of liquid absorbed was compared to the decay-corrected radioactive counts of DTPA absorbed at the same time point. As Figure 4 demonstrates, there was a linear relationship between the amounts of DTPA and liquid absorbed throughout the 24-h test period (*R*^2^ = 0.990, *p* < 0.001). Studies after the addition of 2.5 μL to this cell line demonstrated similar correlations (*R*^2^ = 0.96, *p* = 0.003). Studies after the addition of 10 μL demonstrated correlation but only approached statistical significance (*R*^2^ = 0.56, *p* = 0.09). A study in a different CF cell line (ΔF508/P.11398S) with a 10-μL volume demonstrated significant correlation (*R*^2^ = 0.90, *p* = 0.004).

### The relationship between TER and DTPA absorption

**Figure 5:**
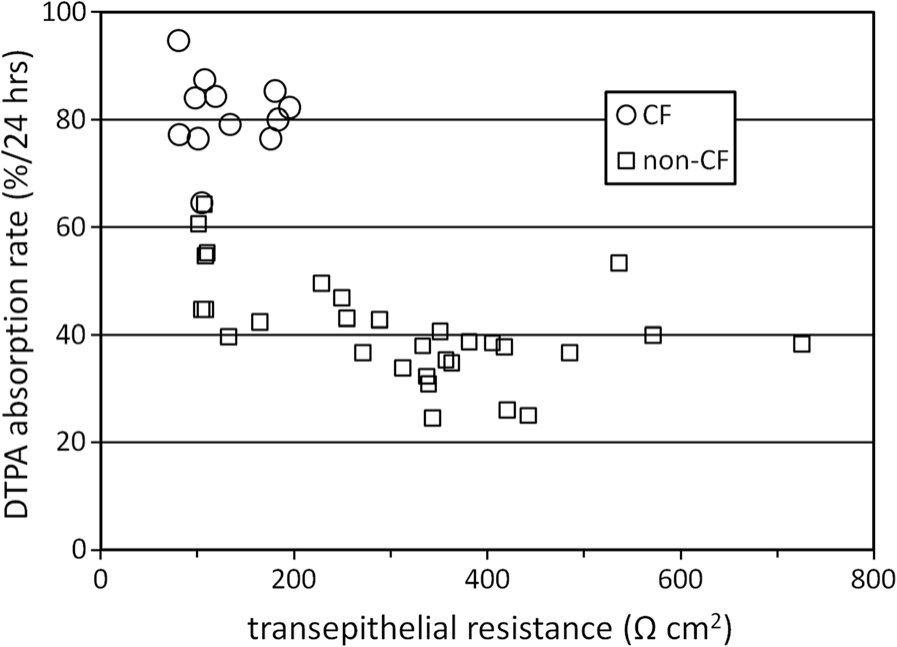
**There is no appreciable correlation between transepithelial resistance and DTPA absorption rate.** Measurements of TER from individual cell cultures were made using a volt-ohm meter 24 h prior to the addition of 10 μL of Tc-DTPA in PBS; one CF line (*n* = 12) and four non-CF lines (*n* = 30).

The measurement of electrical resistance across the airway epithelium is a useful gauge of barrier integrity and tight junction permeability. Previous studies have demonstrated decreased TER in CF epithelial cells [26], so here we sought to determine whether increased DTPA absorption in the CF airway was associated with lower TER and increased tight junction permeability. Figure 5 compares DTPA absorption to TER as measured in individual cell cultures on the day prior to DTPA absorption measurements, using a volt-ohm meter. The average TER was 312 ± 156 and 130 ± 42 Ω cm^2^ for the non-CF (four cell lines, *n* = 30) and CF (one cell line, *n* = 12, ΔF508/ΔF508) cells, respectively (*p* < 0.001 by *t* test). These results indicate significantly lower TER in the CF cells. No relationship between TER and DTPA absorption rate is apparent across an extensive range of TER values. Examining CF and non-CF cultures with TER < 200 Ω also reveals that Tc-DTPA absorption is significantly increased in CF cells (*n* = 12) compared to non-CF cells (*n* = 8) with similar TER levels (*p* < 0.0001). These results do not exclude a role for tight junction permeability in determining DTPA absorption rate, but they indicate that it is likely not the sole cause of the difference between the CF and non-CF cell lines noted here.

### The effects of volume addition on DTPA and liquid absorption

**Figure 6:**
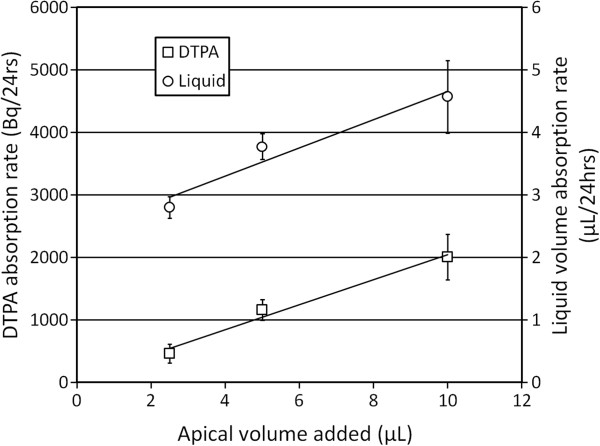
**Liquid and DTPA absorption rates increase linearly with added liquid volume.** Volumes of 2.5, 5, and 10 μL of Tc-DTPA in Ringer’s solution were added to the apical surface of CF HBE cells (one line, six filters per volume), and both DTPA and liquid absorption were measured over 24 h. Each data point is mean ± SEM.

Adding liquid volume to the apical surface of HBE cells has been shown to provide a stimulus for liquid absorption [23]. Figure 6 illustrates that both DTPA and liquid absorption rates increase linearly with added liquid volume. These results indicate a dominant role of liquid absorption in determining DTPA absorption rate in this model.

### DTPA absorption under varying osmotic gradients

**Figure 7:**
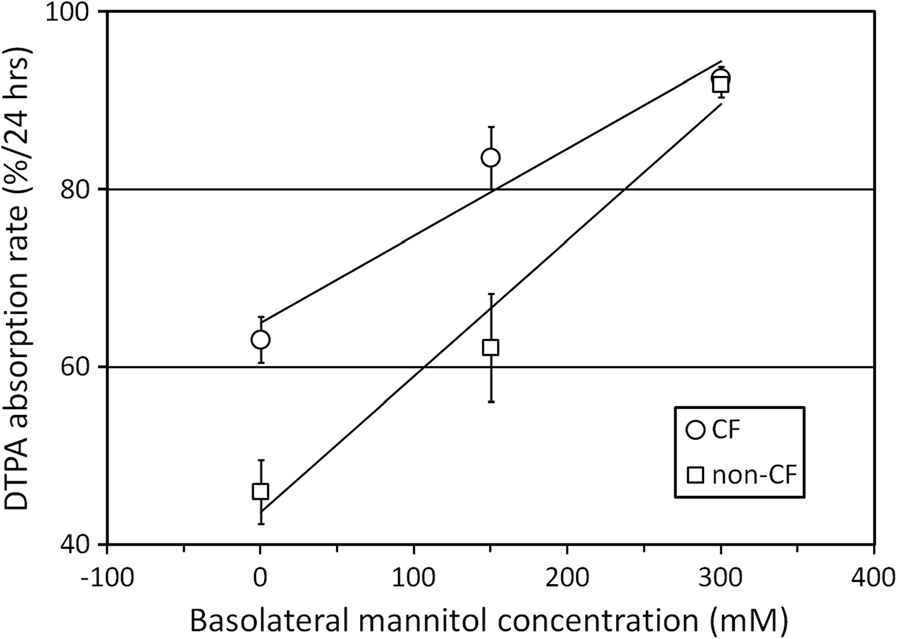
**DTPA absorption is driven by osmotic gradients across the airway epithelium.** DTPA absorption rates were measured in two CF and two non-CF cell lines (*n* = 6 per concentration per line) under increasing basolateral mannitol concentrations. Each data point is mean ± SEM. Measurements were made after the addition of 10 μL of Tc-DTPA in PBS.

To further address the hypothesis that DTPA absorption is directly related to transepithelial liquid movement, osmotic gradients were created to alter liquid movement. Mannitol addition to the basolateral surface of the HBE cultures creates an osmotic gradient favoring liquid absorption. As shown in Figure 7, DTPA absorption rate increased linearly with basolateral mannitol concentration in both CF and non-CF cells, demonstrating a relationship between liquid absorption and DTPA absorption. Two CF (ΔF508/ΔF508, ΔF508/D1152H) and two non-CF cell lines were included. The addition of 300mM apical mannitol, which creates the opposite osmotic gradient, significantly slowed DTPA absorption vs. baseline in CF cells (23.8 ± 10.3%/24 h vs. 41.5 ± 9.1%/24 h; *p* < 0.0001). Previous studies in 16HBE14o- cells demonstrated decreases in TER in response to apical mannitol [27]. To determine whether such changes might play a role in DTPA absorption results, we performed Ussing chamber measurements of TER in non-CF HBEs after the addition of 300 mM mannitol to the apical surface. These studies demonstrated an effect opposite to that previously described. TER increased in response to apical mannitol (baseline TER = 388 ± 93 vs. 622 ± 215 Ω cm^2^, ±SD, *p* < 0.001 by *t* test). This indicates that the alterations in DTPA absorption were not the result of changes in tight junction permeability after mannitol addition.

### DTPA absorption as a gauge of therapeutic response to mannitol

**Figure 8:**
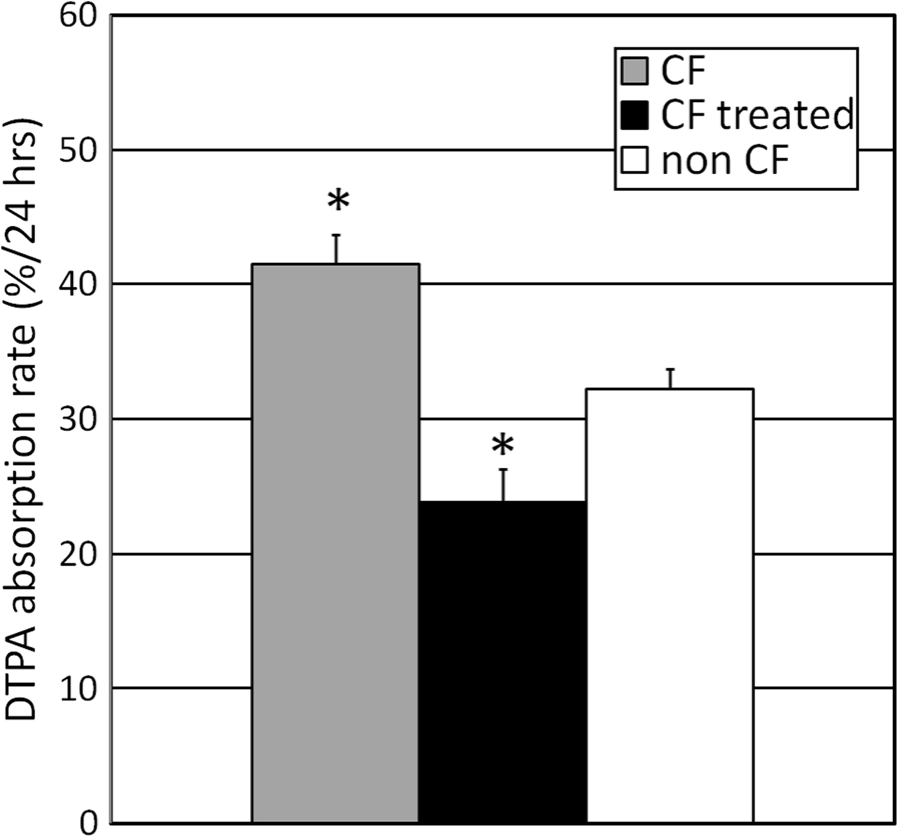
**DTPA absorption in the cystic fibrosis airway is normalized by an apical osmotic gradient.** Apical DTPA absorption was measured in untreated CF epithelial cells and cells with 300 mM mannitol added to the apical surface (three CF cell lines, *n* = 6 cultures per line). Average data from seven non-CF lines presented for comparison purposes. Asterisk, *p* < 0.0001, comparing CF with treated CF. Each data point is mean ± SEM.

Mannitol is currently utilized as an inhaled therapy for CF on the premise that the osmotic gradients created after its deposition in the airways will result in liquid transport from the epithelium into the airway lumen, hydrating secretions and improving mucociliary clearance [25]. As Figure 8 demonstrates, the addition of mannitol to the apical (luminal) surface of the airway epithelium decreases DTPA absorption in CF airway cells (ΔF508/3659delC). This result highlights the relative magnitude of DTPA absorption response compared to baseline measurements in CF and non-CF HBE cultures, and perhaps more importantly, supports the use of DTPA absorption as a marker of liquid movement for therapeutic interventions directed at altering liquid absorption in human airway epithelia.

## Discussion

Liquid transport through the airway epithelium can occur through transcellular and paracellular routes. Rate-limiting ion transport and transmembrane water flux occur through specific ion and aquaporin channels in the cell membrane which restrict the passage of most solutes. Paracellular transport occurs through the tight junctions which are more permeable to small molecule solutes. Transcellular routes are thought to provide the dominant path for liquid absorption based on substantial changes in epithelial cell height observed during apical liquid absorption; however, the relative contributions of these routes have not been definitively determined [21]. Both diffusion and liquid flux may contribute to the paracellular transport of small solutes through the epithelium [19], and a variety of factors related to either or both mechanisms could affect the solute transport rate. A small radiolabeled solute could be used to indicate airway liquid absorption if paracellular liquid flux was significant and proportional to total absorption and if its effect on solute transport was sufficient to allow for measurement. The inhalation of the solute in a liquid aerosol would provide epithelial delivery of both the solute and the liquid volume required to stimulate absorption. Radiolabeled DTPA is a good candidate as a solute in this role based on its safety history and availability. DTPA absorption from the lung has received extensive study, typically as an assessment of whole-lung permeability, and increased DTPA absorption rates have been associated with alveolar damage or increased tight junction permeability resulting from pro-inflammatory stimuli. Our previous studies have demonstrated increased rates of DTPA absorption in the airway-dominated central lung zones of individuals with CF compared to healthy controls [14]. Potential sources of this increased absorption include epithelial damage [16], inflammation-associated increases in tight junction permeability [15], specific effects of CFTR within tight junctions [28,29], and the hyper-absorption of liquid by the epithelium. Lower airway surface liquid volumes in CF could also result in increased concentrations of DTPA in the ASL, potentially contributing to higher rates of concentration-driven diffusion across the epithelium [30].

Here we have used *in vitro* models to better determine whether liquid absorption contributes significantly to increased DTPA absorption in the CF airway and to provide a mechanistic basis for designing a functional imaging method for detecting changes in airway liquid absorption. CF airway cultures absorbed DTPA at a significantly higher rate than non-CF cells, matching the trends of our previous *in vivo* studies. CF airway cultures accurately depict the basic ion transport defects of CF and have been previously shown to hyper-absorb liquid [21]. The epithelial damage that can occur in CF and the pro-inflammatory cytokines associated with infection are not present in this model, though some studies suggest that increased baseline levels of pro-inflammatory cytokines are present in CF HBEs [31,32].

Increased Na^+^ absorption by ENaC has been described as a contributory mechanism driving airway liquid hyper-absorption in the CF airway along with decreased Cl^−^ secretion [2,6,24]. However, a recent study failed to demonstrate differences in Na^+^ absorption between CF and non-CF epithelia [3]. The addition of the long-acting sodium channel blocker benzamil did not affect DTPA absorption rates in our experiments. Our mixed-cell studies demonstrated proportional increases in *I*_Cl−_ and TER and decreases in DTPA and liquid absorption as non-CF HBEs were added to CF HBEs. *I*_Na+_ did not demonstrate a specific trend with cell type in these experiments. Therefore, ENaC-mediated sodium absorption did not significantly influence liquid or DTPA absorption in this specific model.

A linear relationship between DTPA and liquid absorption rates was demonstrated after apical liquid addition to CF HBE cultures. Likely, some portion of this absorption is paracellular, and DTPA is simply conveyed along with liquid flow through the tight junctions between epithelial cells. This result implies that the paracellular movement of DTPA is proportional to the overall liquid absorption by the epithelium when a stimulus for liquid absorption is applied. TER, which provides a means of quantifying tight junction permeability, was lower in the CF cultures when assessed with a volt-ohm meter. There was no relationship between TER and DTPA absorption rate across a large range of TER values, and the DTPA absorption rate was still significantly higher in CF cultures when compared to non-CF cultures with similar TER levels. This result implies that DTPA absorption in these experiments is being driven by factors not directly related to epithelial damage or tight junction permeability. In a setting where no stimulus for liquid absorption is applied, these factors are more likely to influence DTPA absorption rate. TER values increased with the proportion of non-CF cells added to our mixed-cell experiments; however, there was no difference in TER when representative measurements from 9 CF and 11 non-CF cell lines were compared.

Both liquid and DTPA absorption rates increased linearly with liquid volume added to the apical surface of CF HBEs. Previous studies have demonstrated proportional increases in ENaC activity in response to apical fluid addition which provides a stimulus for higher rates of liquid absorption [24]. Though we did not demonstrate a relationship between baseline *I*_Na+_ and DTPA absorption, it is likely that ENaC contributes to liquid absorption when the ASL is expanded. A potential limitation of these experiments is the relative dilution of the added Tc-DTPA solutions by endogenous ASL fluid present in the cultures. This endogenous fluid would have diluted smaller volumes to a larger extent, thus resulting in higher DTPA concentrations after the addition of higher volumes and potentially affecting diffusion-related transport. Mannitol was also utilized to modulate liquid flows through the epithelium in order to demonstrate DTPA absorption response. Basolateral mannitol addition in the HBE cultures establishes an osmotic gradient favoring liquid absorption from the apical (luminal) surface. Our experiments demonstrated that DTPA absorption from the apical surface increased in a dose-dependent manner when mannitol was added basolaterally. Previous studies have indicated decreased TER in association with mannitol [27], but our own Ussing chamber measurements did not support this effect. Both of these experiments suggest that DTPA may provide a surrogate marker for measuring airway liquid absorption; however, it is difficult to assess whether the magnitude of the stimulation used in these *in vitro* experiments is representative of stimuli affecting airway liquid absorption *in vivo*. Such stimuli include the inhalation of a liquid aerosol as previously described [14].

Finally, we considered the potential for DTPA absorption to be used as an indication of therapeutic efficacy. Mannitol has been developed as an inhaled osmotic therapy for use in CF under the premise that it will cause liquid transport into the airway lumen that will hydrate secretions and improve clearance [25]. For our studies, 300 mM mannitol was applied apically along with the Tc-DTPA. Apical mannitol decreased the DTPA absorption rate of CF HBE cells by approximately 40%. This provides an indication that DTPA absorption will provide a robust measure of airway surface liquid modulation *in vivo*. These data also support the validity of DTPA absorption as an acute marker of transepithelial liquid flux. *In vivo* mannitol dosing typically involves a 400-mg inhaled dose. Assuming 50% airway delivery and dilution in 5 ml of ASL, that dose would provide approximately 200-mM mannitol concentrations in the airways. Our dose is therefore of similar magnitude to that used in the *in vivo* setting.

Limitations of the current study include the use of cell culture models which replicate only the epithelial layer of the airway and do not include the vasculature. Blood flow rate through the bronchial artery has been shown to affect DTPA absorption from the airway in animal models [18]. DTPA absorption occurred far more rapidly in our previous *in vivo* studies vs. the current *in vitro* studies (approximately 50%/h vs. 50%/24 h), indicating the potential involvement of other rate-determining factors. Airway cell cultures are the primary vehicle for basic research and therapeutics development in CF in part due to lack of a widely available animal model. In this case, cell cultures provided the best option for relating the basic pathophysiological effects of CF lung disease to the results of our prior imaging studies; however, we cannot assume that the physiological conditions in the cultures are completely representative of *in vivo* conditions. After the start of the experiment, no attempt was made to maintain set DTPA concentrations on the apical surface of the HBE cultures. For this reason, we do not report data in terms of permeability coefficient [33]. Mannitol concentration was similarly allowed to fluctuate after the start of the experiments. The basolateral media volume was significantly larger than the apical volume (10× or more) which would have largely negated the effects of basolateral accumulation of DTPA or mannitol. The limitations of our proposed use of DTPA absorption as an *in vivo* outcome measure lie primarily in the number of factors that could ultimately affect airway permeability to DTPA.

Our studies revealed no direct relationship between TER and DTPA absorption rate *in vitro*, but these experiments all included the application of apical liquid volume which stimulates absorption. It is difficult to assess how the magnitude of our *in vitro* stimuli may relate to those exerted *in vivo*, and other factors involved in epithelial permeability may contribute more substantially to the determination of DTPA absorption rate *in vivo*. It is also possible that different *in vivo* study techniques could be designed to assess these factors individually.

## Conclusions

Establishing a relationship between airway liquid absorption and the absorption rate of an externally detectable radiopharmaceutical would support the development of a functional imaging method for evaluating novel CF therapies. Such techniques could provide for more rapid screening than is currently available from existing outcome measures and biomarkers. Radiolabeled DTPA is attractive for this role based on its availability and safety history in the lung; however, further experiments, particularly demonstration that DTPA absorption reflects a therapeutic response *in vivo*, are needed to determine its utility.

## Competing interests

No authors have any competing interests related to the research presented.

## Authors’ contributions

TEC contributed to the primary study design and interpretation and presentation of data and primarily wrote the manuscript. KMT performed the study and data interpretation. SB contributed to the study design and performed the study and analyzed the data. MMM designed the optical techniques utilized and contributed to the primary development of the HBE model and study design. LWL participated in the manuscript review and revision. JMP contributed to the study design and manuscript review and revision. All authors read and approved the final manuscript.
